# Effects of the Deposition Mechanisms of Silicon Atoms and Tantalum Nanoparticles on the Morphology of Hybrid Films

**DOI:** 10.1021/acsomega.5c08764

**Published:** 2026-01-19

**Authors:** Andrés F. C. Licha, Fábio D. A. Aarão Reis

**Affiliations:** Instituto de Física, 28110 Universidade Federal Fluminense, Avenida Litorânea s/n, 24210-340 Niteroi, RJ, Brazil

## Abstract

Recently fabricated materials with a-Si layers, Ta nanoparticle (NP) scaffolds, and 3.5 atom % Ta/Si ratio have porosity ≈20% larger than that of pure a-Si films and improved performance as anodes of Li-ion batteries. Here, we introduce a model for the growth of these hybrid films that assumes first contact aggregation of NPs deposited with thermal energy and surface diffusion of Si atoms to represent the consequences of their deposition by sputtering. Kinetic Monte Carlo simulations of pure Si films show smooth mounded surfaces with morphology control by Si atom diffusion in convex and flat parts of the surface. Simulations of hybrid films show highly porous Ta NP scaffolds, their partial mixing with Si atoms, and growth of Si clusters separated by nanosized gaps with NPs at their bottom tips. This nontrivial self-organization provides higher porosities and high pore connectivity across the whole film thickness, which is beneficial for ion diffusion. Top views of growing films show widening of Si clusters, in qualitative agreement with scanning electron microscopy images of the previous experimental study. The experimentally observed porosity increase is obtained in simulations with ∼10^4^–10^5^ hop attempts per Si atom and hop probability factors ∼0.03–0.1 for each neighboring atom. Simulated hybrid films are rougher than pure Si films due to fluctuations in the NP heights, which qualitatively agrees with the experiments. Simulations with different thicknesses in each layer but constant Ta/Si ratio lead to lower porosity, which suggests that optimal deposition conditions were chosen in the experiments.

## Graphical abstract

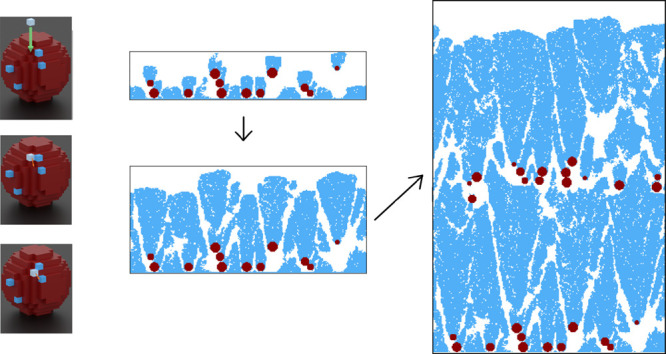

## 1 Introduction

For the improvement of high performance lithium-ion batteries (LIBs), silicon anodes have been widely explored in the last two decades.^1–3^ Some reasons are the high theoretical capacity of silicon for lithium storage, its environmental friendliness, and its abundance in nature.^4^ However, the implementation of Si anodes is difficult because they significantly expand in the lithiation/delithiation process and there may be formation of unstable solid-electrolyte interphases.^4–8^ For overcoming these problems, several technologies were developed to produce Si-based hybrid or composite materials.^6,9–27^

Sputter deposition techniques are frequently used in this field because they form amorphous films, allow the precise control of the film thicknesses, and operate near room temperature, which facilitates the growth on a variety of substrates. A recent example is the use of radio frequency magnetron sputtering for production of 100–200 nm hybrid Si films with Ta nanoparticle (NP) scaffolds on copper foam substrates by Haro et al.^11^ When used as LIB anodes, these films allowed faster Li-ion diffusion and higher charge/discharge speed than pure a-Si films grown with the same method. Despite the small Ta/Si ratio (3.5 atom %), their porosities are ≈20% larger than that of the pure a-Si counterparts. Scanning electron microscopy (SEM) images showed the widening of Si covers around the Ta NPs and atomic force microscopy (AFM) images showed increased surface roughness. Other Si-based composites with comparably high capacities were further developed,^27^ but these hybrid films of Si and Ta NPs are still among the best candidates for improvement of LIBs.^19^

A simple picture to explain the porosity increase in the hybrid films is the stacking layer scheme of Figure 1a, in which a Si layer of low porosity lays above a highly porous Ta NP layer (porosity ∼90%).^11^ However, this picture fails at two points. First, the transport of Li-ions would still be limited by the compact Si layer, which is inconsistent with the faster Li-ion diffusion in the real hybrid films. Second, the scheme neglects the penetration of energetic Si atoms into the NP scaffold and filling of its internal pores, which is physically expected but which may play against the increase of the effective (or connected) porosity.

**Figure 1:**
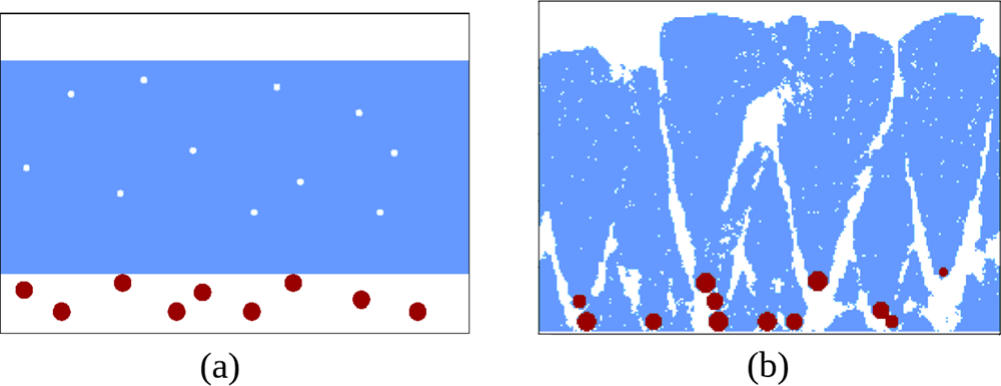
(a) Scheme of the cross-section of a low porosity Si layer above a porous NP layer with total porosity ∼20%. (b) Cross-section of a hybrid film obtained in KMC simulations with similar porosity.

Modeling the deposition of thin solid films has already shown to be an important tool to understand how their physical and chemical properties are related to the growth conditions.^28–31^ Following this reasoning, in this work we introduce a stochastic model for room temperature deposition of Si layers by sputtering and thermal deposition of Ta NP layers with the Ta/Si ratio of 3.5 atom %. The model is built on a lattice where the site volume matches the atomic volume of crystalline Si, so the estimation of characteristic lengths of the films is possible at coarse-grained scales using kinetic Monte Carlo (KMC) simulations. The higher porosity of the hybrid films is quantitatively explained, but our simulations also show an improvement in their morphologies, as illustrated in Figure 1b with one layer of each component. A high mixing of Si atoms in the Ta NP scaffolds is obtained, as expected, but this loss of porosity is compensated by the formation of nanoscale gaps between the Si clusters that grow with NPs at their bottom tips. Those gaps connect the pore system across the whole film thickness, implying a high effective porosity that facilitates diffusive transport across the layers. The hybrid film simulations also show widening of the top parts of the Si clusters as they grow and shows the increase of the roughness, both features in qualitative agreement with the experiments. The model also help us to explain why the layered deposition improves these films and why the choice of the layer thicknesses in the experiments of Haro et al.^11^ was optimal.

Previous models of nonreactive growth by sputtering assumed that collective diffusion of the adsorbed atoms (adatoms) was the main relaxation mechanism,^32–34^ possibly including effects of the impact of energetic species.^35^ That assumption is the same of models of homoepitaxial deposition of metals and semiconductors from vapors with thermal velocities, which is described by the interplay between surface diffusion and deposition flux.^29–31,36^ However, this type of relaxation is expected to be slow at room temperature, which was the condition in the deposition experiments. For this reason, the model proposed here approximates the effects of the high kinetic energy of a Si atom that reaches the film surface by the transient diffusion of this atom in a quenched surface landscape, i.e. neglecting the collective relaxation of the previously deposited atoms.^37,38^ The deposition of low energy Ta NPs is represented by their aggregation at the first contact with the growing film, which promotes the formation of porous deposits similarly to ballistic deposition models.^39–43^

The rest of this paper is organized as follows. Section 2 presents the deposition model with its physical interpretation, the quantities to be calculated, and information on the simulation method. Section 3 separately presents results of simulations of pure Si films; results of simulations of hybrid films with a focus on the model conditions that match the experimental porosities; extension of the simulations to Si and Ta layers with different thicknesses, which suggest that the experiments were performed in the optimal conditions for the given Si/Ta stoichiometry. Section 4 discusses the relevance of the model for deposition by sputtering, which also has potential application to other materials, and discusses the relations between the simulated hybrid films and the experimental results. Section 5 summarizes our results and conclusions.

## 2 Model and Methods

### 2.1 Deposition Model

The films are grown in simple cubic lattices with site edge *a* = 0.272 nm. The corresponding volume of a site, 0.0201 nm^3^, is the atomic volume of a Si crystal at room temperature and normal pressure. This lattice-based model can be a reasonable approximation for amorphous films at coarse-grained scales, which roughly are length scales ≳3 nm (i.e., 10 times larger than the site edge or more). In this approximation, the interaction of an atom with the lattice neighbors represents the average interaction of an atom in the amorphous sample. Similar assumptions formerly justified the use of a lattice model to describe the kinetics of plasma enhanced chemical vapor deposition of amorphous carbon–nitrogen films.^44^

The deposition occurs on a flat substrate located at *z* = 0 and substrate atoms are assumed to be immobile. Periodic boundary conditions are considered in the *x* and *y* directions of the simulation cell whose lateral length is denoted as *L*. The set of adatoms with the same (*x*,*y*) position is termed a column of the deposit and the height variable *h*(*x*,*y*) is the *z* coordinate of the topmost occupied site in that column (it may occupied by a Si adatom or by a site of a NP).

The two species of the model are illustrated in Figure 2a: each Si atom occupies one lattice site and each Ta NP is a discretized sphere whose diameter is of 11 lattice units. This NP size roughly corresponds to the average NP diameter of 3 nm used in the production of the hybrid films.^11^ The size dispersion of the NPs in that experiment was not negligible, but our assumption of a single size is justified as a first step to explain the nontrivial interplay of the two species in a hybrid film.

**Figure 2:**
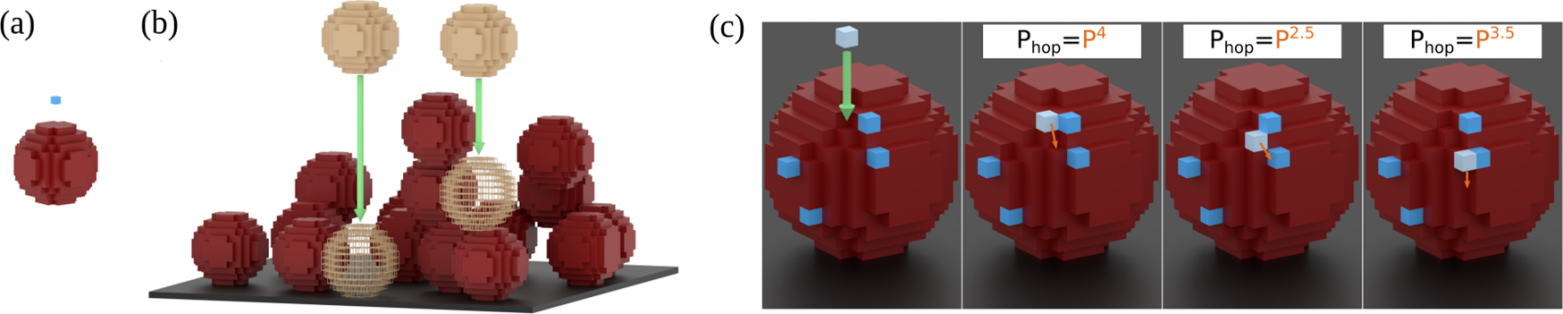
(a) Si atom (blue) and Ta NP (red) in the lattice-based model. (b) Incidence of two NPs (light brown) that aggregate at the first encounter (translucent positions) with previously aggregated NPs (red). (c) Incidence of a Si atom (lightest blue) that aggregates at the first contact with the deposit and its three first hops (orange arrows) with the corresponding probabilities *P*_hop_. Dark blue cubes are previously aggregated Si atoms.

Considering the density 16.65 g/cm^3^ of crystalline Ta, each NP has approximately 8 × 10^2^ Ta atoms (this is not the number of sites of the discretized sphere because a Ta atom does not occupy the same average volume of a Si atom in a crystal). The numbers of deposited atoms and NPs are chosen to fit the Ta/Si ratio of 3.5 atom % of the experiments:^11^ since the number of Ta atoms in a scaffold corresponds to a compact Ta film thickness 1.2 nm, each scaffold is built with deposition of 0.0061 NPs per column (i.e., per substrate site); after deposition of a scaffold, a Si layer is built with the sequential deposition of 135 Si atoms per column. A sequence of 5 Ta NP scaffolds and 5 Si layers are deposited.

The dynamics of aggregation of NPs and Si atoms are separately described in the next subsections.

#### 2.1.1 Deposition of Ta NPs

During the deposition of a scaffold, the NPs are sequentially released with their centers at randomly chosen (*x*,*y*) and with their lowest sites at positions *z* larger than the maximal height of the deposit. Each NP moves in the −*z* direction (toward the substrate) and permanently aggregates at the first contact with a nearest neighbor (NN) occupied site, which may be a site of the substrate or of the deposit. The process is illustrated in Figure 2b. The aggregation at the first contact is assumed because the experiments of interest were performed with no potential bias for NP deposition, which led to soft landing with an estimated energy smaller than 0.1 eV.^11,45^ Deformations of the deposited NPs are also neglected.

This model resembles the so-called ballistic deposition (BD).^39^ BD of single site particles with aggregation only to NNs produce deposits with porosity ∼70%,^43^ while aggregation to NNs or NNNs leads to porosities >90%.^46^ BD of larger spherical particles with aggregation to NNs and off-lattice BD lead to porosity ∼85%.^40,42,47^ For these reasons, the first contact aggregation of Ta NPs is expected to produce highly porous scaffolds.

#### 2.1.2 Deposition of Si Atoms

During the Si layer deposition, each atom is released at a position *z* larger than the maximal height of the deposit, moves in the −*z* direction, and is adsorbed at the first contact with an occupied NN site (left panel of Figure 2c). As the atom reaches the film surface or the substrate, the spread of its kinetic energy leads to a temperature increase in the neighborhood of the landing position. However, for simplicity, the model assumes that only the last deposited atom is mobile, while the other atoms of the film have frozen positions.

The relaxation of a Si atom is represented by a sequence of *S* hop attempts to randomly chosen neighboring sites and permanent aggregation after the last attempt. The value of *S* is expected to increase with the energy of the incoming atom, i.e. the larger this energy the longer the atom can move. This is reasonable because the spread of a larger thermal energy in the atom collision is expected to lead to a longer time for the neighborhood of the collision point to return to the room temperature (in which the atom mobility is expected to be negligible).

Each hop is executed only if two conditions are fulfilled, otherwise the atom remains in the current position. First, the hop has a probability *P*_hop_ that depends on the local surface morphology, as defined below. Second, a target site for the hop is randomly chosen among the 6 NN sites and the 12 NNN sites, with probability 1/18 for each of them, and the hop to the target site is allowed only if it has at least one occupied NN. The second condition prevents desorption of the deposited atoms.

The hops of the Si atom are assumed to be thermally activated and the activation energy is assumed to increase with the local curvature of the interface; indeed, larger curvatures are typically related to higher bonding with the rest of the deposit.^48,49^ This assumption is similar to that of thin film growth models with collective adatom diffusion.^50^ To minimize the effect of the lattice structure, the local curvature is written as the isotropized discrete Laplacian of site occupation, $\frac{1}{6}\left(2\sum_{\mathrm{NN}}N_{i}+\sum_{\mathrm{NNN}}N_{j}-24\right)$ , where *N*_*i*_ = 1 (0) for each occupied (empty) NN and *N*_*j*_ = 1 (0) for each occupied NNN.^51^ Excluding additive constants, the activation energy can be written as

$$E=E_{0}\left(\sum_{\mathrm{NN}}N_{i}+1/2\sum_{\mathrm{NNN}}N_{j}-1\right)$$ 1

where *E*_0_ > 0. With this form, *E* = 0 for an atom in the configuration of minimal bonding, with 1 occupied NN and no occupied NNN, while *E* > 0 in other configurations. The hop probability is then written as

$$P_{\mathrm{hop}}=P^{\left(\sum_{\mathrm{NN}}N_{i}+1/2\sum_{\mathrm{NNN}}N_{j}-1\right)}$$ 2

where

$$P=\exp\left(-\frac{E_{0}}{k_{B}T}\right)$$ 3

*k*_B_ is the Boltzmann constant, and *T* is the temperature in the region where the kinetic energy of the incoming Si atom was redistributed. Thus, *P*_hop_ = 1 for an atom in the configuration of minimal bonding and *P*_hop_ < 1 in other configurations.

The first three executed hops of a mobile Si atom are illustrated in Figure 2c and the corresponding probabilities *P*_hop_ are shown. For simplicity, the interaction energy *E*_0_ does not distinguish the three species involved in the problem (Si, Ta, and substrate); this is assumed because most relevant interactions during the deposition are those between Si atoms, which comprise 96.5 atom % of the deposit.

The dimensionless parameters *S* and *P* are taken as the two free model parameters in this work. These parameters will be interpreted in terms of surface diffusion lengths of Si atoms when the morphology of the film surfaces is analyzed. However, we cannot relate these parameters to microscopic interactions because they can hardly be estimated from the complex dynamics of sputter deposition; the same occurs with the (alternative) parameters *E*_0_ and *T*. For instance, the high kinetic energy of the Si atom is redistributed in a small region around the landing position, so *T* is expected to be much higher than the room temperature, but it would be very difficult to estimate the size of that region and the time for its convergence to room temperature.

The mechanism of Si atom diffusion considered here is frequently termed transient relaxation or limited mobility^37,38^ because the adsorbed atom moves during a short time interval and becomes immobile after that time, i.e., it is frozen when its neighborhood returns to room temperature. This contrasts with models for deposition on substrates at high temperatures, which consider simultaneous diffusion of all surface atoms.^29–31,36^ Other models with transient adatom relaxation were formerly applied to thermal vapor deposition^37,38^ and electrodeposition^52,53^ because they stabilize energetically favorable structures at short length scales. However, the porous deposits obtained here are very different from the deposits obtained in those applications (e.g., dendritic films in electrodeposition models and compact films in vapor deposition models).

### 2.2 Quantities of Interest

The film thickness *H* is the average value of the height variable,

H=⟨h̅⟩ 4

where the overbar denotes a spatial average over the columns {*x*,*y*} and the angular brackets denote an average over different configurations with given numbers of deposited atoms and NPs. The surface fluctuations are characterized by the film roughness

$$\begin{array}{cc} W=\sqrt{W_{2}}=\langle ({\overset{¯}{\mathrm{h̃}}}^{2})\rangle^{1/2}, & \mathrm{h̃}\equiv h-\overset{¯}{h} \end{array}$$ 5

The inner part of the deposits is characterized by the total and the effective porosities, which are defined below.

Each deposit is limited by six surfaces: the substrate (*z* = 0), four lateral sides, and the top outer surface formed by the sites with *z* = *h*(*x*,*y*) (i.e., the topmost occupied sites of each column). The total volume of the deposit is *V*_T_ = *L* ^2^ *H* (see eq 4). The number of occupied sites is denoted as *N*_d_ (which may contain Si atoms or Ta NPs), so the volume occupied by the deposit is *N*_d_ *a* ^3^; those sites are indicated in red in the two-dimensional scheme of Figure 3. The remaining sites form the pore volume; in Figure 3, they are marked in white and yellow. The total porosity ϵ_T_ is defined as the pore volume fraction below the top outer surface:

$$\epsilon_{T}=\frac{V_{T}-N_{d}a^{3}}{V_{T}}=1-\frac{N_{d}a^{3}}{V_{T}}$$ 6

**Figure 3:**
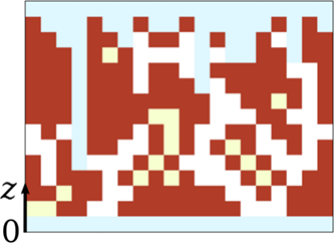
Two-dimensional scheme showing the solid part of a deposit (red), connected pores (white), isolated pores (yellow), and the two reservoirs (light blue). Pore connectivity in this scheme assumes periodic boundaries in the lateral directions.

The effective porosity ϵ_E_, which is also called connected porosity, is the fraction of the volume *V*_T_ that can be filled by a fluid transported from a reservoir at the substrate (*z* = 0) and a reservoir formed by all empty sites above the top outer surface [all points with *z* > *h*(*x*,*y*)]. These reservoirs are shown in light blue color in Figure 3. The connected pore system is defined as the set of empty sites with 0 < *z* < *h*(*x*,*y*) that are connected to both reservoirs by some sequence of NN empty sites (white in Figure 3). Denoting the volume of this system as *V*_c_, the effective porosity is

$$\epsilon_{E}≡\frac{V_{c}}{V_{T}}$$ 7

The remaining empty sites (yellow in Figure 3) form the isolated pores.

### 2.3 Simulation Details

One Si layer comprises the deposition of 135­(*L*/*a*)^2^ atoms; a compact crystalline film with the same mass has thickness of ≈37 nm. One NP scaffold comprises the deposition of 0.006­(*L*/*a*)^2^ Ta NPs, so that the ratio between the numbers of atoms in a Ta NP scaffold and in a Si layer is 3.5 atom %, as reported in the experimental study of the hybrid films.^11^

Pure films with five layers of Si (no scaffold) were simulated with parameters in the ranges 10^2^ ≤ *S* ≤ 10^5^ and 0.001 ≤ *P* ≤ 0.1. Hybrid films with five Ta NP scaffolds and five Si layers were grown with the same parameters, starting with the deposition of a scaffold. The roughness and the total porosity were calculated in short thickness intervals during the deposition. The most time-consuming calculation of the effective porosity was performed only at the end of the deposition of each layer using a three-dimensional version of the Hoshen–Kopelman algorithm.^54,55^

In order to check the suitability of changing the deposited masses in each scaffold and in each layer, we also performed deposition of hybrid films with those masses multiplied by some factor *M* while keeping the Si/Ta ratio constant. This is equivalent to changing the nominal thickness of each scaffold and each layer by the factor *M*. The factors *M* = 1/2, 2, and 3 were considered.

Simulations with the above parameters were performed in lattices with lateral size *L* = 512*a* ≈ 139 nm. Some simulations in lattices with *L* = 2048*a* ≈ 557 nm were performed to confirm that there is no relevant finite-size effect in the average quantities.*l* They were implemented with in available computers with Threadripper 2970WX and Ryzen 5950X processors.

## 3 Results

### 3.1 Simulations of Pure Si Deposition

#### 3.1.1 Overview of the Film Morphology

Figure 4a–d shows top views and vertical cross-sections of the Si films grown in simulations with four pairs of parameters (*S*,*P*). All images are obtained after the deposition of five Si layers, so they contain the same number of atoms (675 atoms per substrate site). Figure S1 of the Supporting Information shows views of Si films grown with other values of *S* and *P*.

**Figure 4:**
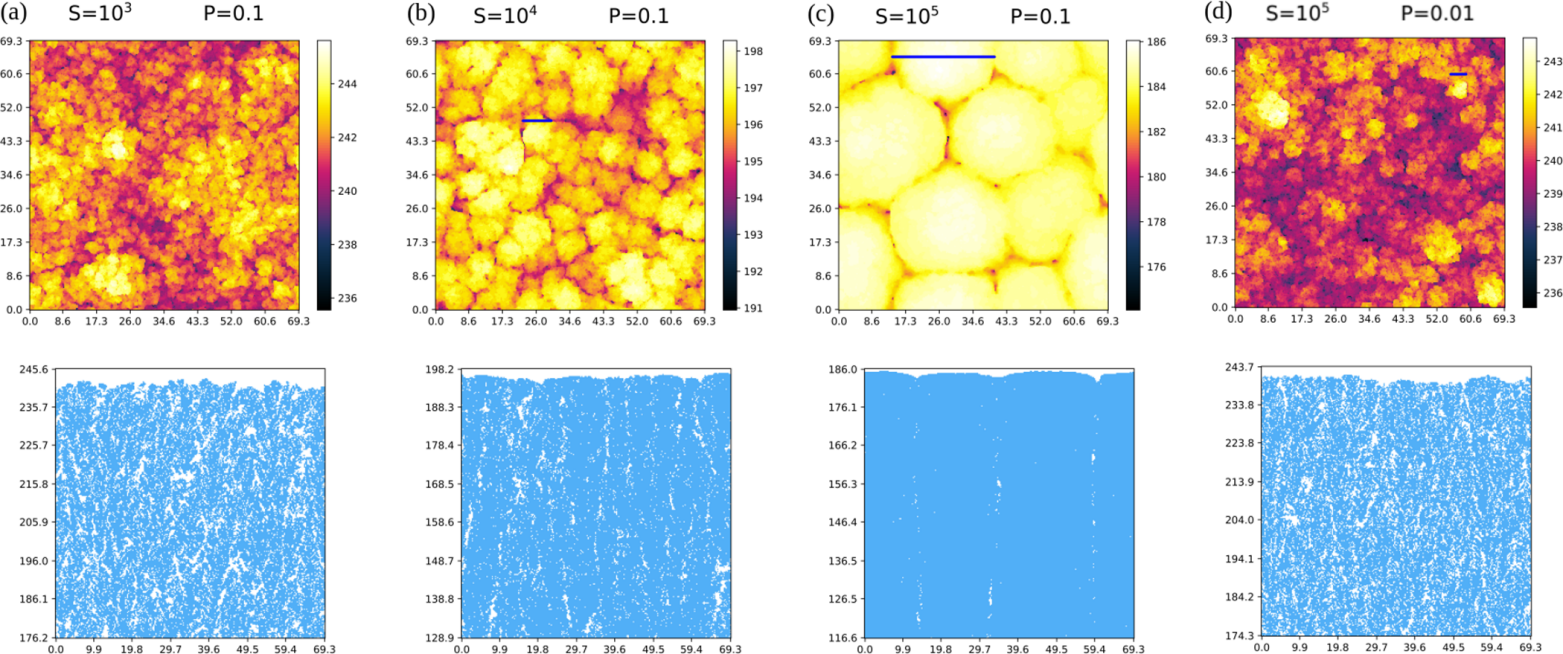
(a–d) Top and cross-sectional views of pure Si films grown in simulations with the indicated parameters. All lengths are in nanometers. In (b–d), blue bars indicate typical widths of surface mounds.

The films grown with the smallest values of *S* and *P* are highly porous because the aggregation to lateral NNs and the small mobility facilitate pore formation; see Figure S1 with *S* = 10^2^ and *P* = 0.001. Indeed, the growth without surface diffusion (*S* = 0) is equivalent to the ballistic deposition model, in which the aggregation occurs at first contact and the porosity is ∼70%.^43^ When *S* increases and *P* is constant, the porosity decreases, as observed for *P* = 0.1 and *S* varying from 10^3^ to 10^5^ in Figure 4a–c. The same occurs when *P* increases and *S* remains constant, as observed for *S* = 10^5^ and *P* varying from 0.1 to 0.001; Figures 4c,d and S1. The diffusion allows the atoms to reach positions below the first contact, where they can find more stable positions because the numbers of NNs and NNNs are larger.

The surfaces of the deposits have mound patterns when grown with *P* = 0.1 and *S* ≥ 10^3^ or with *P* = 0.01 and *S* ≥ 10^5^; their typical widths are indicated in Figure 4b–d and in one panel of Figure S1. These mounds are narrow and have disordered edges for the smallest *S* and *P*, but for the largest values of those parameters they are wide, have smooth and rounded borders, and are separated by narrow gaps only a few micrometers deep (e.g., Figure 4c). The largest mounds have an approximated hexagonal organization, which shows that the model of adatom relaxation partly overcomes the effects of the simple cubic geometry used in the simulations. This morphology resembles those of the a-Si films of Haro et al.^11^ and of sputter deposited films of other materials.^15,26,56–60^ However, it differs from the morphologies obtained in previous models of deposition by sputtering;^32–34^ see discussion in Section 4.1.

The comparison of Figure 4c,d also shows that the decrease of *P* by a factor 10 leads to a significant decrease of the porosity, which may also be achieved by decreasing *S* by a factor ∼10^2^ (Figure 4a,c). This anticipates that the parameter *P* has a more important effect on the porosity than *S*.

#### 3.1.2 Porosity and Roughness

Figure 5a,b shows the total porosity and the effective porosity as a function of the thickness in films grown with *P* = 0.03 and 0.1, respectively, for several values of *S*. Figure S2 of the Supporting Information shows the same quantities in films grown with smaller values of *P*.

**Figure 5:**
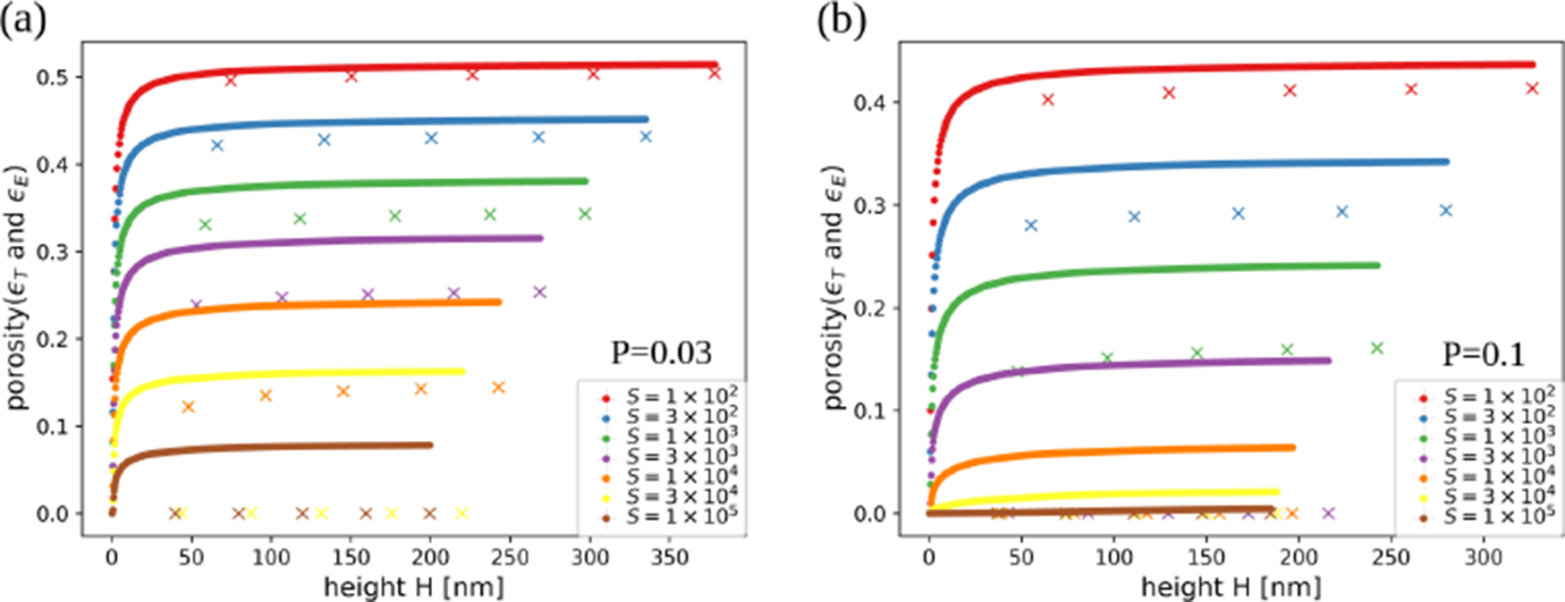
Evolution of total porosity and effective porosity with the thickness of films grown in simulations with (a) *P* = 0.03 and (b) *P* = 0.1 for the indicated values of *S*. The uncertainties in the porosity are nearly of the same order as the size of the data points.

For all parameter sets, the effective porosity is smaller than the total porosity, which shows that isolated pores are present. For *P* ≥ 0.03, the largest values of *S* lead to ϵ_E_ = 0, so there are only isolated pores. Observe that these porosities account only for pores of the size of a site or larger, so the simulated deposits with vanishing effective porosity do not exclude connectivity at length scales smaller than the site edge. This is the case of Li^+^ ions, whose effective diameter is ≈0.15 nm^61^ (nearly half of the lattice constant of the model). Thus, due to the limitations of the lattice model to represent subnanometer features, the possibility of transport of these ions across the film is not excluded.

Figure 6a,b shows the surface roughness as a function of the thickness in films grown with *P* = 0.01 and 0.1, respectively, for several values of *S*. Figure S3 of the Supporting Information shows the same quantity in films grown with other parameters. The general trend is that the roughness decreases as *S* or *P* increases, which shows a smoothening effect of the adatom relaxation. Similar effect is observed in growth models with collective adatom diffusion with thermally activated rates.^38^ However, in all cases the roughness is small (nanoscale roughness), typically varying from ≈0.5 nm (for the largest *S* and *P*) to ≈1.5 nm (for the smallest *S* and *P*).

**Figure 6:**
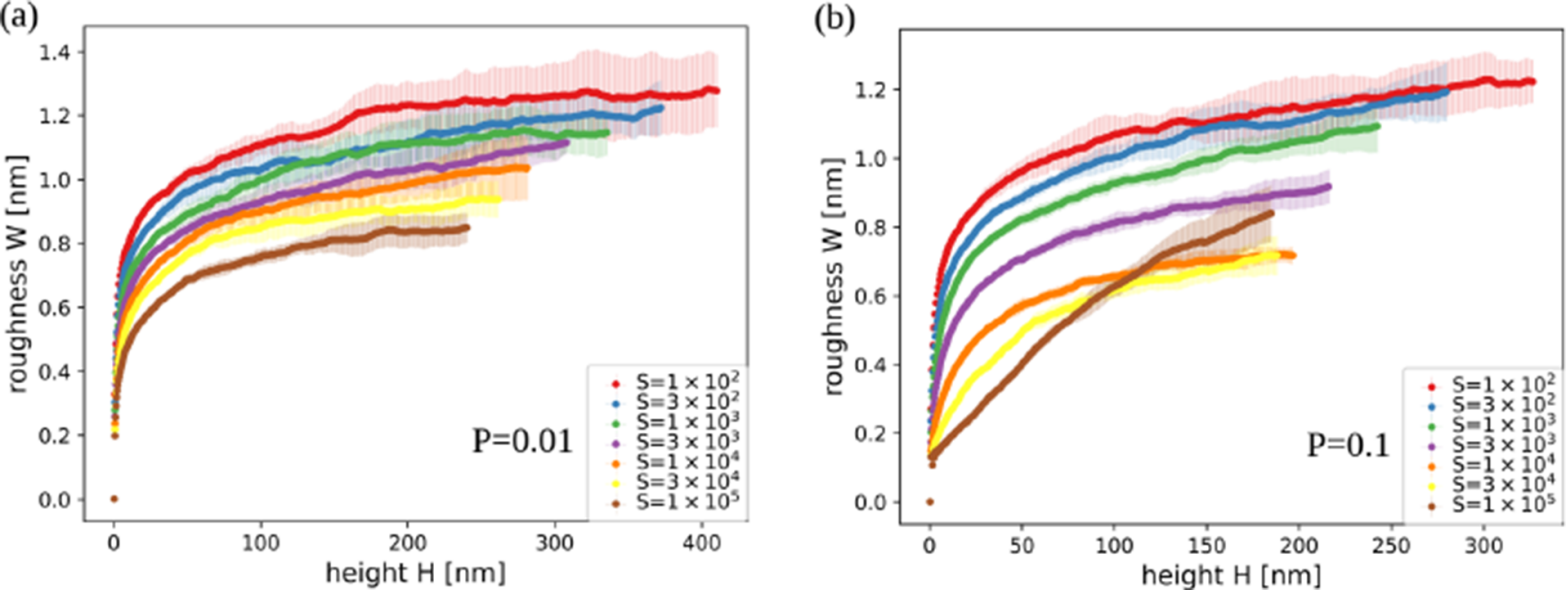
Surface roughness as a function of the thickness of films grown in simulations with (a) *P* = 0.01 and (b) *P* = 0.1 for the indicated values of *S*. The shadowing shows the uncertainties obtained from averaging over different samples.

The experimental work of Haro et al.^11^ shows a-Si films with low roughness and rounded mounds, which are qualitatively similarly to those obtained in our simulations with *S* ≳ 10^3^ and *P* ≳ 0.03. These a-Si films have poor performance as anodes, which suggests slow Li^+^ ion diffusion in their pores. For this reason, we understand that the simulated films with nearly zero effective porosity provide the best qualitative representation of those experiments. This is achieved with *S* ≥ 3 × 10^4^ for *P* = 0.03 and with *S* ≥ 3 × 10^3^ for *P* = 0.1. Thus, modeling of the hybrid films will be restricted to these parameter ranges.

#### 3.1.3 Scaling Relations

If the Si atom moves in a landscape where the hop probability is *P*_hop_ = *P* ^*k*^ (i.e., the term inside the brackets of eqs 1 and 2 is equal to *k*), then the number of executed hops is of order *SP* ^*k*^. The number of hops is expected to affect the morphology; e.g., a large number of executed hops is expected to help the formation of low energy surfaces with large areas.

The porosity is one of the morphological quantities that should be affected by changes in the average numbers of executed hops. For this reason, we plotted the porosity (total and effective) as a function of *SP* ^*k*^ for several values of *k* to search for the value that provides the best data collapse in the low porosity region (typically ≲0.3); in this search, the porosity measured at the final configurations was considered. The best data collapse was obtained for both porosities with *k* = 2, so Figure 7a shows them as a function of the scaling variable *SP* ^2^.

**Figure 7:**
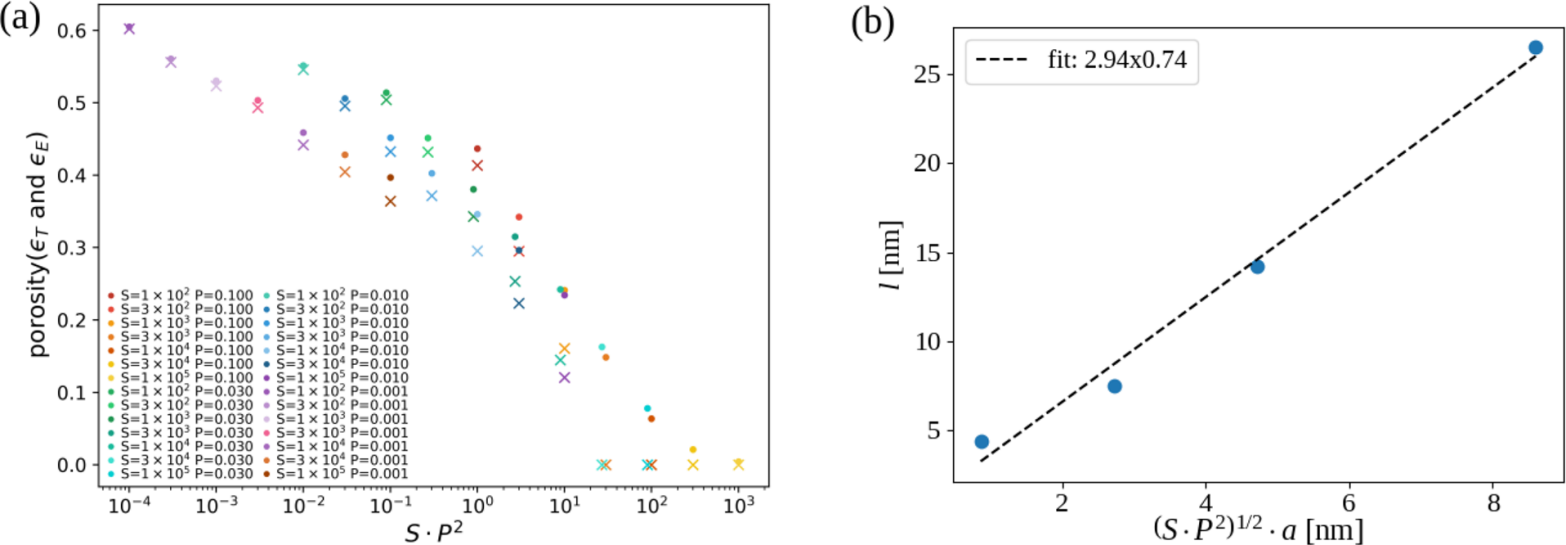
(a) Total (circles) and effective (crosses) porosities as a function of the scaling variable *SP* ^2^ for the indicated sets of parameters. (b) Mound width *l* as a function of the diffusion length (*SP* ^2^)^1/2^ *a* associated with that scaling variable.

The value *k* = 2 may represent sites with 3 NNs and no NNN; 2 NNs and 2 NNNs; 1 NN and 4 NNNs. They are sites at convex regions of the surface or on a flat terrace. The data collapse in Figure 7a indicates that the diffusion in these configurations controls the film morphology; since *SP* ^2^ ≳ 1 in the region where the collapse is obtained, the Si atom can execute at least some steps in those configurations or in configurations with less neighbors. Moreover, since *P* ≤ 0.1 was considered in our simulations, the Si atoms will be effectively immobile in configurations with more neighbors and lower energy (e.g., sites in the middle of flat planes or at their straight borders).

The interpretation of *SP* ^2^ as an average number of hops suggests that Si atoms have diffusion lengths of order (*SP* ^2^)^1/2^ *a* because the average length of a hop is of the same order of the lattice constant. The formation of mounds in the film surfaces is expected to be a consequence of Si atom diffusion, so the average mound width *l* is expected to be proportional to that diffusion length. Rough estimates of the mound widths are obtained from the lengths of the blue bars in the top views of the films in Figures 4b–d and S1. Figure 7b shows these widths as a function of the diffusion length and a linear fit that confirms their proportionality. Thus, the mound widths of real films deposited by sputtering are expected to be on the same order of magnitude of the diffusion lengths of the atoms during their relaxation.

We did not try to relate the roughness with *SP* ^2^ because the roughness obtained in our simulations is ≲1.5 nm and features with such small lengths scales are not expected to follow scaling laws (which are defined at coarse-grained level).

### 3.2 Simulations of Hybrid Film Deposition

#### 3.2.1 Hybrid Film Structure

Figure 8a–d shows vertical cross-sections and top views of the simulated hybrid films with one Ta NP scaffold and some fractions of a Si layer. The scaffold is formed by sparsely distributed NPs and has high porosity because each NP aggregates at the first contact with another NP (or with the substrate); a contact between two of the NPs can be seen at the right side of the cross-sectional views. The fluctuations in the heights of NP aggregation are also large.

**Figure 8:**
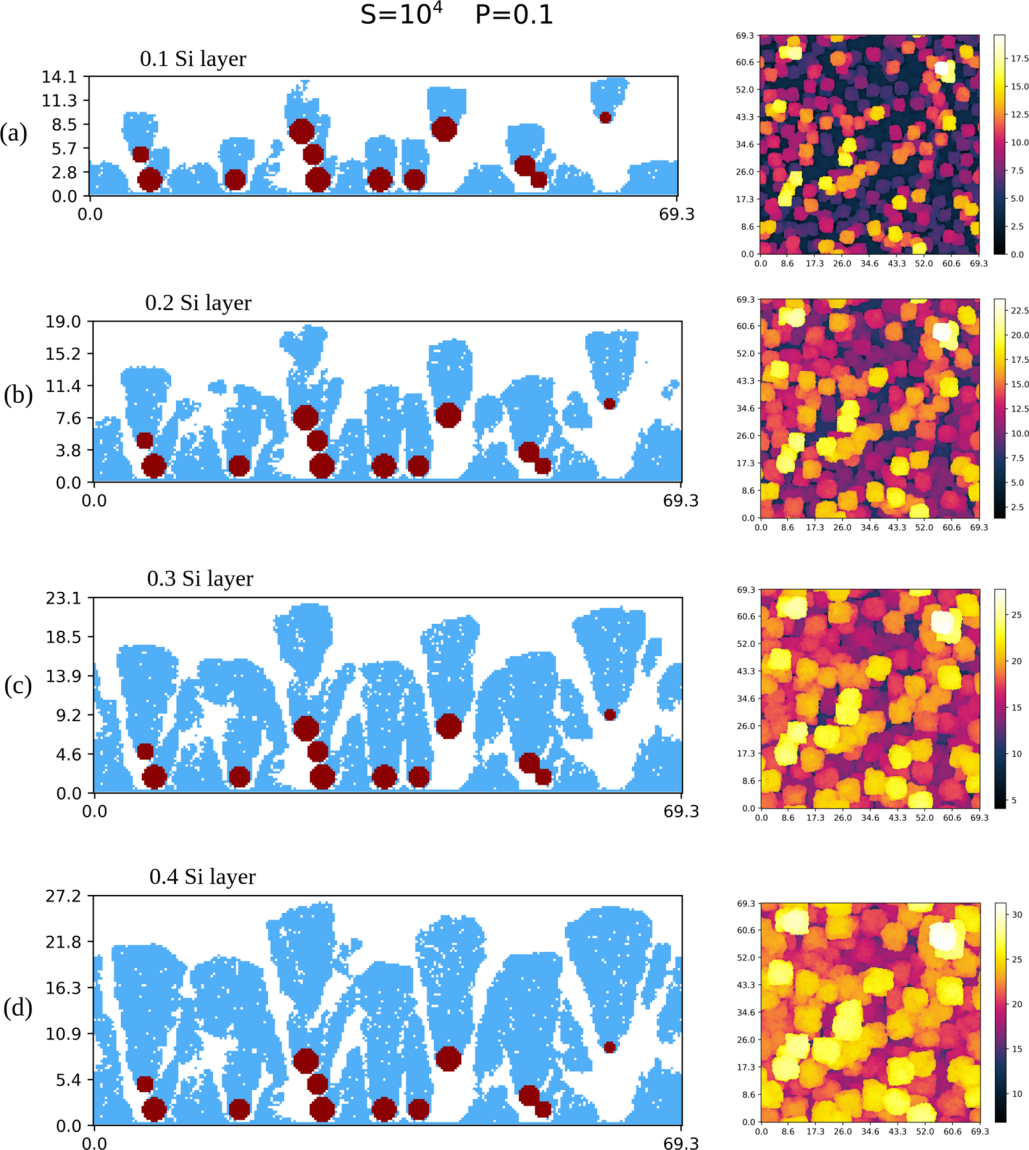
(a–d) Cross-sectional and top views of hybrid films grown in simulations with *S* = 10^4^ and *P* = 0.1 after the deposition of the first Ta NP scaffold (red in cross-sectional views) and the indicated fractions of a Si layer (blue in cross-sectional views). All lengths are in nanometers.

Many Si adatoms aggregate on exposed parts of the substrate and form clusters with disordered shapes there. The penetration of these Si atoms in the NP scaffold rules out the simplified two-layer picture of Figure 1a. However, most of the Si atoms form clusters on the top of the NPs; these clusters grow vertically and widen in the horizontal directions. The fluctuations in the NP heights lead to large fluctuations in the thickness of the hybrid film. After deposition of 0.2 Si layer, no additional deposition on the clusters formed on the substrate is observed, confirming that most Si atoms aggregate at the topmost parts of the film. The cross-sections of some Si clusters show an approximately conical shape with the NPs at their vertices, but the top parts of these apparent cones are rounded. The Si clusters also have isolated pores similar to those of the pure Si films grown with the same model parameters, *S* = 10^4^ and *P* = 0.1; see Figure 4b.

Figure 9a–e shows a sequence of vertical cross-sectional views of a film grown with the same parameters after the deposition of each pair of Ta NP scaffold and Si layer. The Ta NP scaffolds are porous, but the NPs are highly mixed with the Si atoms. When a Si layer is growing and widening, the topmost clusters capture most of the incoming atoms and prevent the growth of the clusters with lower heights (shadowing effect). This leads to the formation of nanosized gaps between the clusters, which visually have a large contribution to the total porosity. These gaps also contribute to the pore connectivity in the vertical direction.

**Figure 9:**
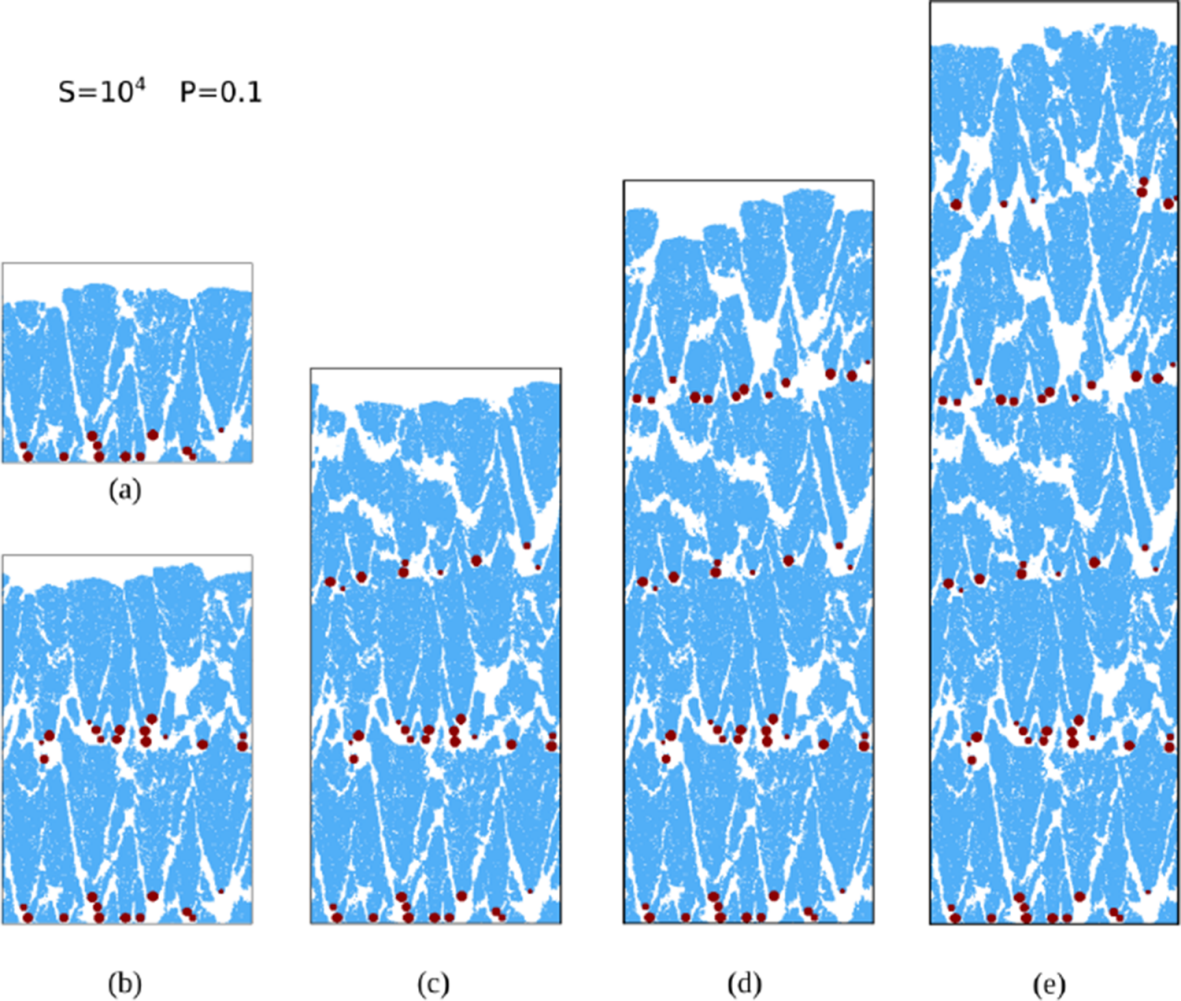
Cross-sectional views of hybrid films grown in simulations with *S* = 10^4^ and *P* = 0.1 after the deposition of each pair of a Ta NP scaffold and a Si layer: (a) 1 pair; (b) 2 pairs; (c) 3 pairs; (d) 4 pairs; and (e) 5 pairs. The horizontal size of each image is 69.6 nm.

Top views of the same films after the deposition of 1 and 5 pairs of scaffolds and layers are shown in Figure 10a,b, respectively. The widths of the top parts of the Si cones do not have significant changes, which indicates that the morphology is renewed after the deposition of each pair.

**Figure 10:**
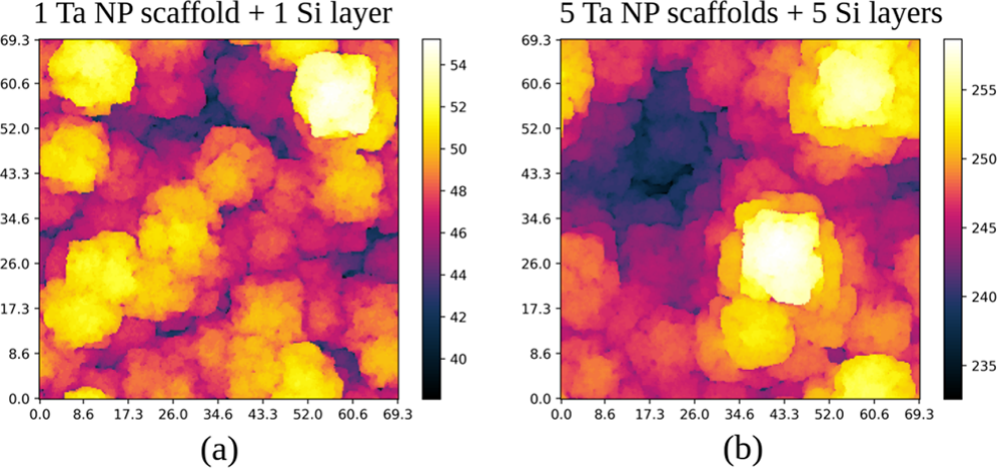
Top views of hybrid films grown in simulations with *S* = 10^4^ and *P* = 0.1 after the deposition of (a) 1 and (b) 5 pairs of Ta NP scaffolds and Si layers (the same films whose cross-sections are in Figure 9a,d, respectively). All indicated lengths are in nanometers.

Figure 11a,b shows vertical cross-sectional views of films with five pairs of scaffolds and layers grown with other values of *S* (3 × 10^4^ and 10^5^) and with *P* = 0.1. The main features of the NP scaffolds and Si clusters are the same, but the comparison with Figure 9e shows that the increase of *S* produces less porous Si clusters. Moreover, the gap widths are visibly narrower for *S* = 10^5^ (Figure 11b), which anticipates a decrease in the effective porosity; see Section 3.2.2.

**Figure 11:**
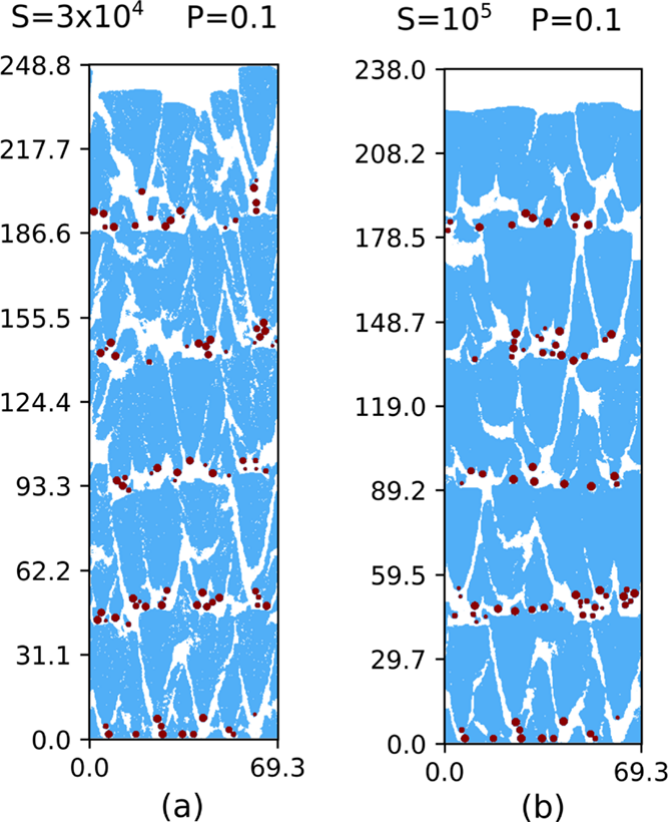
Cross-sectional views of hybrid films with 5 Ta NP scaffolds and 5 Si layers grown in simulations with *P* = 0.1 and: (a) *S* = 3 × 10^4^; (b) *S* = 10^5^. All indicated lengths are in nanometers.

Figures S4 and S5 of the Supporting Information show that the morphologies of films grown with *P* = 0.03 and 10^4^ ≤ *S* ≤ 3 × 10^4^ are similar to those described above. The main difference is the higher internal porosity of the Si clusters, consistently with the previously reported effect of *P* on pure Si films.

#### 3.2.2 Porosity and Surface Roughness

Figure 12a,b shows the porosities ϵ_T_ and ϵ_E_ as a function of the thickness *H* in simulated hybrid films with *P* = 0.03 and 0.1, respectively, for several values of *S*. For *S* = 10^2^ (the smallest *S*), the two porosities have small differences because most Si atoms aggregate at the first contact or after a small number of hops, so that most of the randomly distributed pores are connected to the top and to the bottom surfaces (similarly to ballistic deposition^43^). Small differences between ϵ_T_ and ϵ_E_ are also obtained for *S* ≥ 3 × 10^4^ and *P* = 0.1, whose cross-sections are shown in Figure 11a,b. This result quantitatively shows that most nanosized gaps between the Si clusters contribute to the connected porosity, while the density of internal pores of those clusters is relatively small.

**Figure 12:**
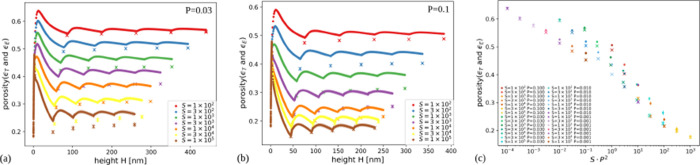
(a, b) Total porosity and effective porosity as a function of the thickness of hybrid films grown in simulations with *P* = 0.03 and *P* = 0.1, respectively. The values of *S* are indicated in the plots. (c) Total (circles) and effective (crosses) porosities as a function of the scaling variable *SP* ^2^ for the indicated sets of parameters.

Figure 12a,b also shows oscillations of the total porosity, which increases when a NP scaffold is being deposited and decreases during the Si layer deposition. This is expected because the Ta NP deposition produces highly porous scaffolds (estimated as ∼85% for ballistic deposition of spheres^40^) and the Si deposition produces films with very low porosity for large *S* and *P* (Figure 5b). The oscillations are large when the first scaffold grows, but they do not exceed 5% in the other scaffolds because the Ta/Si ratio is low and the Ta NPs are highly mixed with the Si atoms.

Figure 12c shows the porosities of the final hybrid film configurations (i.e., after deposition of 5 pairs of scaffolds and layers) as a function of *SP* ^2^. Similarly to the case of pure Si films, for *SP* ^2^ ≳ 1 and porosities ≲0.4, there are good collapses in curves characteristic of each porosity. This shows that the film morphology is controlled by the diffusion of Si atoms on the same typical surface configurations observed in the pure Si films, despite the quantitative change in the porosities due to the NP scaffolds.

Figure 13a,b shows the evolution of the surface roughness *W* with the thickness *H* for the same parameters of Figure 12a,b, respectively. In all samples, the roughness rapidly increases during the deposition of a NP scaffold and decreases when a Si layer is deposited. The consequent roughness oscillations have amplitudes 1–2 nm. After deposition of five pairs of scaffolds and layers, a final roughness of 4–5 nm is attained in all cases, with weak dependence on the parameters *P* and *S* that control the Si aggregation. The roughness of the hybrid films is much larger than that of the simulated pure Si films (Figure 6a,b). This large roughness is mostly an effect of the fluctuations in the positions of NP aggregation, as anticipated by visual inspection of the initial stages of the growth of a Si layer (Figure 8a–d).

**Figure 13:**
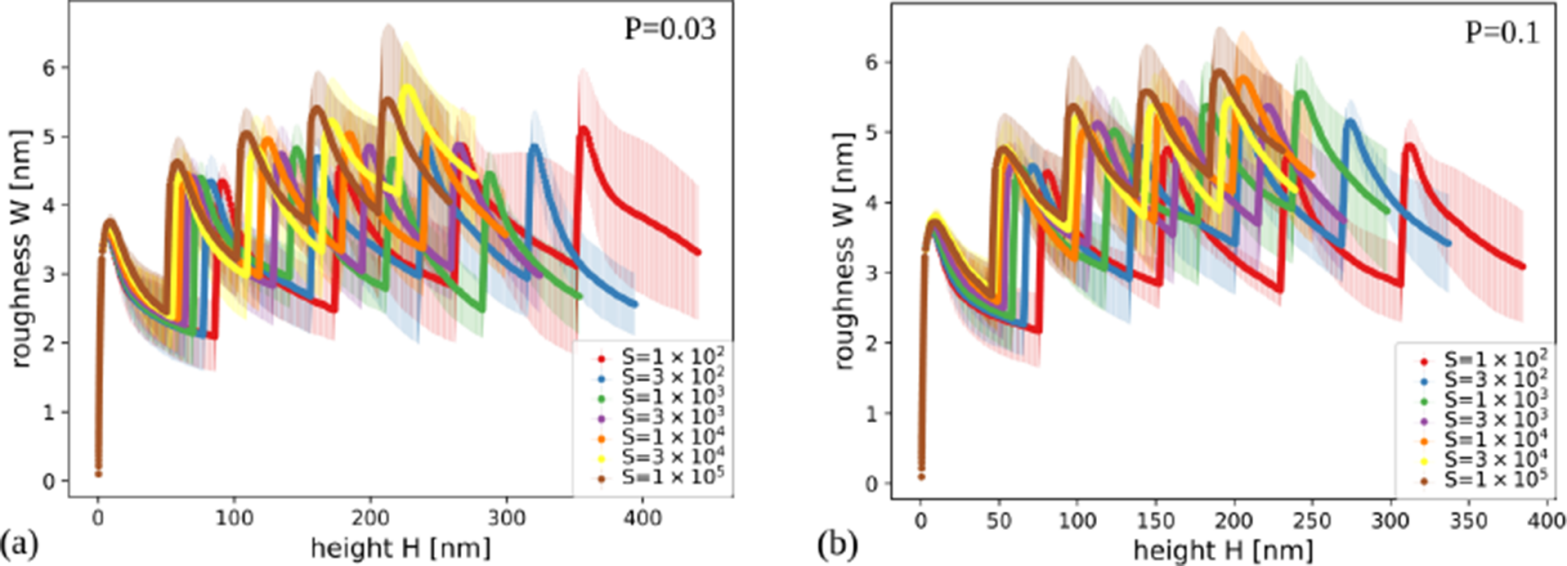
Surface roughness as a function of the thickness of films grown in simulations with (a) *P* = 0.03 and (b) *P* = 0.1. The values of *S* are indicated in the plots and the shadowing indicates the uncertainties in the roughness.

#### 3.2.3 Films with Other Thicknesses of Ta NP Scaffolds and Si Layers

Here we show results of simulations of hybrid films with different thicknesses of the Ta NP scaffolds and of the Si layers, but with the 3.5 atom % Ta/Si ratio kept constant. In each case, the deposited mass of each scaffold and each layer is multiplied by the factor *M*, but the total mass of the films are approximately the same (this is achieved by dividing the number of deposited scaffolds and layers by, roughly, the same factor *M*).

Figure 14a,b shows the evolution of the effective porosity in films grown with (*S* = 10^4^, *P* = 0.1) and (*S* = 10^5^, *P* = 0.03), respectively, for *M* = 1/2, 2, and 3. The data for *M* = 1, which are the same of the previous simulations, are also shown for comparison. For *M* < 1 or *M* > 1, the changes lead to films with smaller effective porosities.

**Figure 14:**
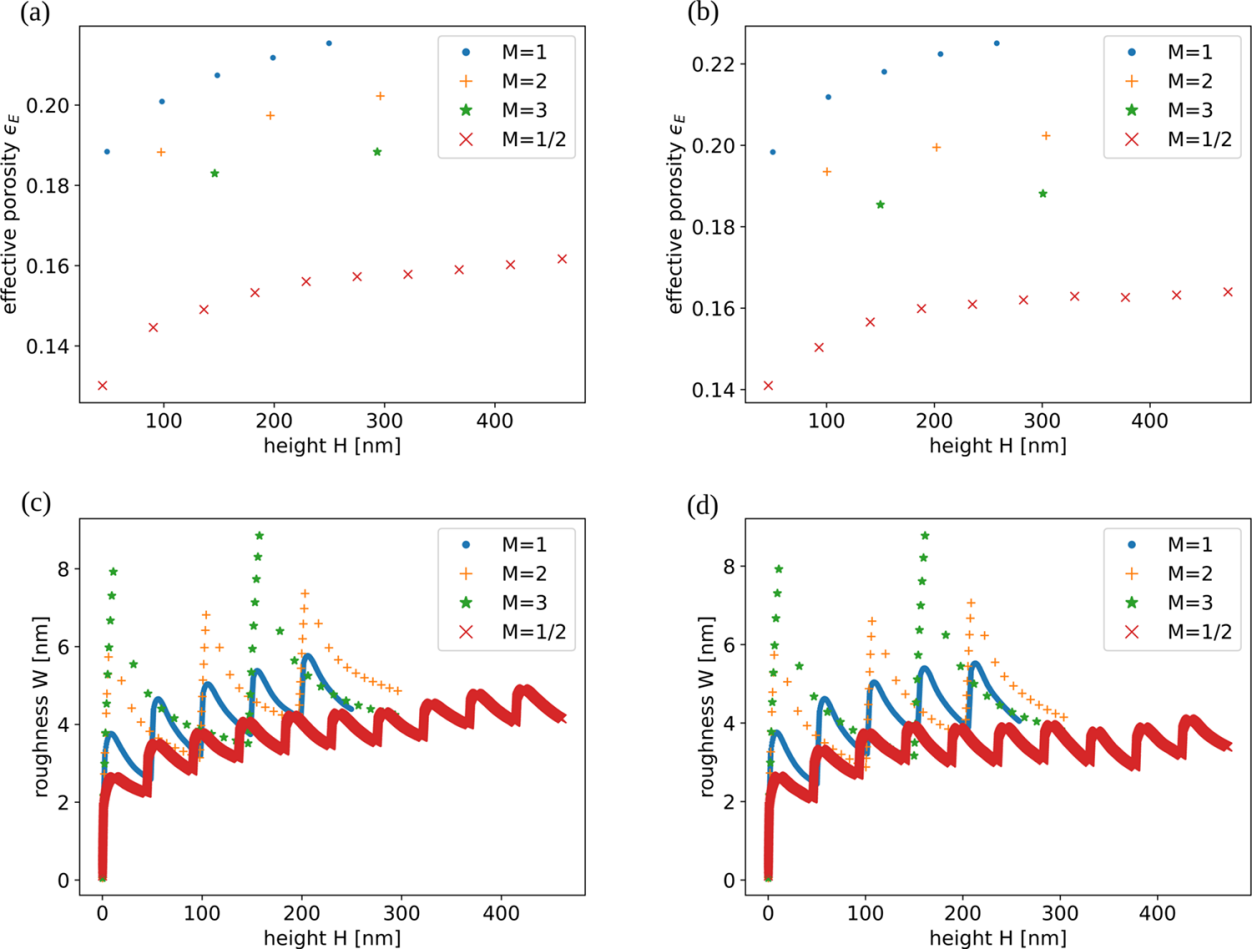
(a) Effective porosity as a function of the thickness of films grown in simulations with *S* = 10^4^, *P* = 0.1, and the indicated factors *M* for multiplying the masses deposited in each Ta NP scaffold and each Si layer. (b) The same for *S* = 10^5^ and *P* = 0.03. (c) Surface roughness as a function of the thickness of films grown in simulations with *S* = 10^4^, *P* = 0.1, and the indicated factors *M* for multiplying the masses deposited in each Ta NP scaffold and each Si layer. (d) The same for *S* = 10^5^ and *P* = 0.03.

Figure S6 of the Supporting Information shows cross-sections of films grown with those values of *M*. When *M* = 1/2 is considered, the scaffolds are less thick than those of *M* = 1, so they have smaller pore density, while the gaps between the Si clusters have approximately the same width. For *M* = 2 and 3, the scaffolds have larger pores, but the larger masses of each layer lead to narrower gaps between the Si clusters and some of these gaps may be blocked; this explains the smaller effective porosity in comparison with *M* = 1 (Figure 14a,b). These results show that the thicknesses of scaffolds and layers that matched the experimental values (*M* = 1)^11^ were the best choices to improve the porous media for the given Si/Ta ratio.

Figure 14c,d shows the surface roughness evolution of the films grown with the same parameters above and the same changes in the masses of scaffolds and layers. For *M* = 1/2, the roughness is slightly smaller than that of films with *M* = 1, but it is still much larger than that of the pure Si films (Figure 6a,b). For *M* = 2 and 3, the roughness is larger. The roughness peaks are obtained after the deposition of each scaffold, which confirms that the Ta NPs are responsible for the larger roughness when compared to the pure Si films.

## 4 Discussion

### 4.1 Application of the Sputter Deposition Model

The model of thin film deposition by sputtering assumes that the relaxation of an atom occurs in a short time interval after its adsorption due to the temperature increase in a region of nanoscopic size near the collision point. That increase is caused by the dissipation of the kinetic energy of the incident atom. As an approximation, the position of the other deposited atoms are assumed to be frozen during the relaxation. The model does not include collective diffusion of the adsorbed atoms because it is designed for low temperature deposition, which is the case of room temperature for many materials. The assumption of transient relaxation warrants that the nanopatterns produced by the model are stable. The model was defined on a simple cubic lattice where the site volume matches the atomic volume of a Si crystal (site edge 0.272 nm), so the estimation of some characteristic lengths of the grown films (at a coarse-grained scale) is possible in the simulations.

Most simulations of pure Si films with thickness ∼200 nm and parameters *S* ≳ 10^3^ and *P* ≳ 0.03 produced surfaces with roughness on the order of 1 nm and rounded mounds whose widths were a few nanometers or more (Figure 4a–c). Although an atomistic interpretation of two model parameters is difficult, their combined effect on the film morphology can be quantitatively determined. The scaling of the porosities (total and effective) and of the mound widths in terms of these parameters indicate that the film morphology is controlled by the transient diffusion of the Si atoms in convex or flat surface configurations. The mound widths are of the same order of magnitude as the diffusion lengths involved in those scaling relations, which are expected to represent the average diffusion lengths of the sputtered atoms after reaching the film surface.

In the study of sputtered a-Si films of Haro et al.,^11^ scanning electron microscopy (SEM) images showed rounded mounds, but with apparently higher dispersion of mound widths than those shown in our simulations. Atomic force microscopy (AFM) of the film surfaces showed roughness 5.82 nm. The sputter deposition of other materials also produces surfaces with nanoscale roughness and wide rounded mounds (on the order of tens or hundreds of nanometers). Some examples are TiO_2_ films on glass (reactive sputtering),^56^ layered Si–Ni films on Cu foils,^15^ layered electrodes with TiO_*x*_, Ag, and Al-doped ZnO on polymeric substrates,^57^ amorphous multilayers of Ge and C on a Cu substrate,^26^ AlN films on different substrates (reactive sputtering),^62^ ZnO films doped with Mg and F on glass,^58^ CdS films on a fluorine-doped tin oxide coating,^59^ and CdMgZnO films with different Cd concentrations.^60^ This suggests that the model may be extended to other sputtered deposited materials.

Notably, previous sputter deposition models considering collective adatom diffusion led to different morphologies: disordered rough surfaces in models designed for amorphous materials;^32,33^ faceted or columnar morphologies in models designed for crystalline materials.^34^ However, within these models, surface nanopatterns may be unstable due to the simultaneous diffusion of all Si atoms at the substrate temperature. For these reasons, the present model is a reasonable first step to represent the experimentally deposited a-Si films, despite the different values of the roughness.^11^

The quantitative discrepancy in the roughness may have several origins; some possibilities are the window sizes used in the roughness measurements, the roughness of the Cu foam substrates used in the experiments, and the assumptions for the atomic flux. First, the simulations are performed on a flat substrate and the maximal cell width is 139 nm, while the AFM images of the a-Si films had width ∼1 μm and showed large domains of hills and valleys, each one containing several small rounded mounds.^11^ Second, the Cu foam substrate has 120 pores per inch^63^ and this substrate disorder may contribute to fluctuations in the film thickness. Third, the small roughness of the simulated films may be partly attributed to the collimated atom flux that facilitates filling of surface gaps. A mixed advective and diffusive atom flux is expected to increase the roughness, as shown in other deposition models.^52,64^ However, taking into account all these factors would require much superior computational capabilities than those currently available.

### 4.2 Application of the Hybrid Film Model

Haro et al.^11^ presented SEM images of hybrid films with one NP scaffold covered by nominal Si thicknesses of 3 and 15 nm. The images showed an increase in the width of surface waves as the Si coverage increased, reaching widths of ∼20 nm; the authors suggested the formation of radial coatings of Si around the NPs. The top views of simulated films in Figure 8a–d are consistent with the widening of the surface ondulations during the deposition of a Si layer. However, the cross-sections show that the waves are the top parts of Si clusters with NPs at their bottom tips. Detailed inspection of the images of simulated films (Figures 9a–e and 11a,b) show that Si coatings are formed below some NPs, but they are atomically thin.

The experimentally estimated porosity of the hybrid films is 15–20% larger than that of the pure a-Si films. This difference was calculated from the film thicknesses, so it is a difference of total porosities (not effective porosities). The simulations give differences of total porosities in this range for the following parameters: (*P* = 0.03, 3 × 10^4^ ≤ *S* ≤ 10^5^) and (*P* = 0.1, 3 × 10^3^ ≤ *S* ≤ 10^5^) (Figures 5a,b and 12a,b). The effective porosity of the pure a-Si films grown with these parameters is zero (Figure 5a,b), i.e. the pure Si films only have isolated pores. However, it is important to stress that our approach considers connectivity of NNs on a lattice where the minimal pore size is the diameter ≈ 0.3 nm of a Si atom, so the model cannot exclude connectivity of narrower pores in the amorphous films produced in the experiments (which allow transport of Li^+^ ions). This is an additional reason for our comparison of porosity differences (from pure Si to hybrid films) instead of a comparison of absolute values of the porosities.

The experimentally deposited hybrid films had roughness 10.83 nm, which was significantly larger than the roughness of 5.82 nm of the pure a-Si films.^11^ This result is also in qualitative agreement with our simulations; Figures 13a,b and 6a,b. The simulations show that the roughening is a consequence of the fluctuations in the heights of NP aggregation, which in turn is a consequence of neglecting their surface diffusion (due to their low incoming energy). Possible reasons for the quantitative differences are those discussed in the context of pure a-Si film growth (Section 4.1).

Observe that the picture that emerges from our model, as synthesized in Figure 1b, is consistent with an increase of pore connectivity across the whole film thickness. In applications of the hybrid films as LIB anodes, this distribution of wide pores is essential for faster Li-ion transport across the films, as observed in the experiments.^11^ Importantly, the improved film morphology is a consequence of a realistic description of the deposition kinetics in our model: the penetration of the fast Si atoms into the scaffolds reduces the initially high porosity of that region, but the large Ta NPs are the basis for the growth of separated Si clusters and formation of vertically connected pores in the Si layers. Instead, the series association of two layers suggested in Figure 1a would not have this benefit because the high porosity would be restricted to the Ta NP scaffolds and the rate of ion transport would be limited by the compact Si layer.

Another important issue of Si-based anodes is their swelling during the lithiation/delithiation process and the consequent decrease in cyclability. This issue was partially overcome in the hybrid films of Si and Ta NPs when compared to pure a-Si films.^11^ The qualitative picture obtained in our simulations (Figure 1b) suggests that this improvement may result from the distribution of nanopores across the whole film thickness, which can accommodate approximately uniform expansions of the material. This interpretation differs from that of Haro et al, who claim that the NP scaffolds create heterogeneities in the stiffness that prevent volume expansions.^11^ A study of the mechanical properties of the model films under electrochemical operation would be necessary to discriminate between these proposals, but such a study is out of the scope of the present work.

As a final note, in another recent work, the same authors performed deposition of hybrid films with Si flux at an acute angle.^16^ In this case, TEM images showed a columnar structure in which the Si clusters grew vertically and laterally after covering the Ta NPs. Our simulations predict a similar morphology, but they consider a flux perpendicular to the substrate, so it is not possible to anticipate the quantitative changes that might appear in simulations with a flux in acute angle.^65^

### 4.3 Effect of the Layer Thicknesses

To understand the relevance of the layered deposition for improving the hybrid film morphology, we begin with a discussion of two limiting cases that are not beneficial for the application in LIBs.

First, if the two species are simultaneously deposited, we expect that the Ta NPs will be disperse in a compact Si film due to the small fraction (3.5 atom %) of the former. The film features will be dictated by the dynamics of the Si atoms (the majority of the atoms) when they reach the film surface, so the morphology will be similar to that of pure Si films. In our model, this would be the case *M* → 0 in Section 3.2.3, i.e. a vanishingly small mass in each layer.

Second, if the layers are extremely thick, then the simple picture of Figure 1a is expected. Mixing of Si atoms and Ta NPs will be restricted to a thin interface between two thick stacking layers. The film will have a high total porosity, but the diffusion across the film will be slow because it will be limited by the compact Si layer. In our model, this would be the case *M* → *∞* in Section 3.2.3.

This analysis shows that an improved morphology is expected for some finite thicknesses of the Ta NP scaffolds and of the Si layers. In our model, this is expected for some finite *M* for the given stoichiometry. Our simulations for several *M* show that the experimental values of those thicknesses, which correspond to *M* = 1, lead to the highest effective porosities in the above range of parameters *S* and *P*. This feature is expected to be beneficial for Li-ion transport in the connected pore system. Thus, our results suggest that Haro et al.^11^ have chosen the best conditions for deposition of the hybrid films under the constraint of constant 3.5 atom % Ta/Si ratio. Indeed, fine-tuning of the growth conditions is common in high quality experimental work.

## 5 Conclusions

We performed KMC simulations of models for deposition of a-Si films by sputtering and for deposition of hybrid films in which Si layers are intercalated with porous scaffolds with ∼3 nm Ta NPs deposited with thermal velocities. The study is inspired in recent experiments^11^ and the main aim is to understand the increase in porosity and pore connectivity of the hybrid films when compared with the a-Si counterparts.

The model considers transient diffusion of the Si atoms after adsorption due to the dissipation of their high kinetic energy. As an approximation, we neglect the relaxation of other atoms in the region near the point of collision of the Si atom with the deposit; the rest of the deposit remains frozen to account for the room temperature condition of the substrate. The simulations of pure Si films show smooth film surfaces with rounded mounds, which are in qualitative agreement with those experiments and which resemble the morphologies of surfaces of other materials deposited by sputtering techniques. To our knowledge, this morphology was not obtained in previous models of thin film deposition by sputtering. The diffusion of the Si atoms at convex and flat surface configurations is shown to be the process controlling the film morphology; the mound width is shown to be of the same order of magnitude as the diffusion length of these atoms.

The model of Ta NP deposition considers first contact aggregation due to their low kinetic energy. The simulations of hybrid films are performed with the same deposited thicknesses of Ta NP scaffolds and Si layers of the experiments, corresponding to a 3.5 atom % Ta/Si ratio.^11^ In suitable ranges of parameters, the simulations show Si clusters growing with the NPs at their bottom tips and nanosized pores formed in the NP scaffolds and between the Si clusters. This grants a high connected porosity across the whole thickness of the hybrid films, which is beneficial for ion transport. The morphology of the hybrid films is controlled by Si diffusion in the same configurations observed in the pure Si films, despite the much larger porosity of the former. We obtained a range of model parameters that quantitatively match the change in the experimentally measured porosity,^11^ while the larger surface roughness of the hybrid films could be described only qualitatively.

In this suitable range of model parameters, simulations were also performed with smaller and larger thicknesses of Ta NP scaffolds and Si layers, but keeping constant the 3.5 atom % Ta/Si ratio. These simulations reveal a decrease in the effective porosity in both cases, which shows that the choice of the thicknesses in the experimental work were the best conditions consistent with the given stoichiometry. Thus, the modeling approach may be helpful to anticipate the optimal growth conditions for other hybrid films where porosity and connectivity are important properties.

## Supplementary Material



## Data Availability

The data shown in this paper is available at https://github.com/andresfernando-git/Modeling-the-Deposition-of-Hybrid-Films-with-Silicon-Layers-and-Tantalum-Nanoparticle-Scaolds

## Notes

Additional images of deposits grown in simulations and additional plots of porosity and roughness (PDF)
