# Supplementary material for: Effects of the Deposition Mechanisms of Silicon Atoms and Tantalum Nanoparticles on the Morphology of Hybrid Films
Source: ACS Omega. 2026 Jan 19;11(4):5436–49. doi: 10.1021/acsomega.5c08764 (PMC12878341; doi:10.1021/acsomega.5c08764)
Supplement: Supplementary file 1 [file ao5c08764_si_001.pdf]

Supporting Information
Effects of Different Deposition Mechanisms of Silicon and Tantalum Nanoparticles on the Morphology of Hybrid Films

Andrés Fernando Cardozo Licha
Fábio D. A. Aarão Reis

Instituto de Física, Universidade Federal Fluminense, Avenida Litorânea s/n, 24210-340 Niterói, RJ, Brazil
fdaar@protonmail.com

June 26, 2025

Pure Si films

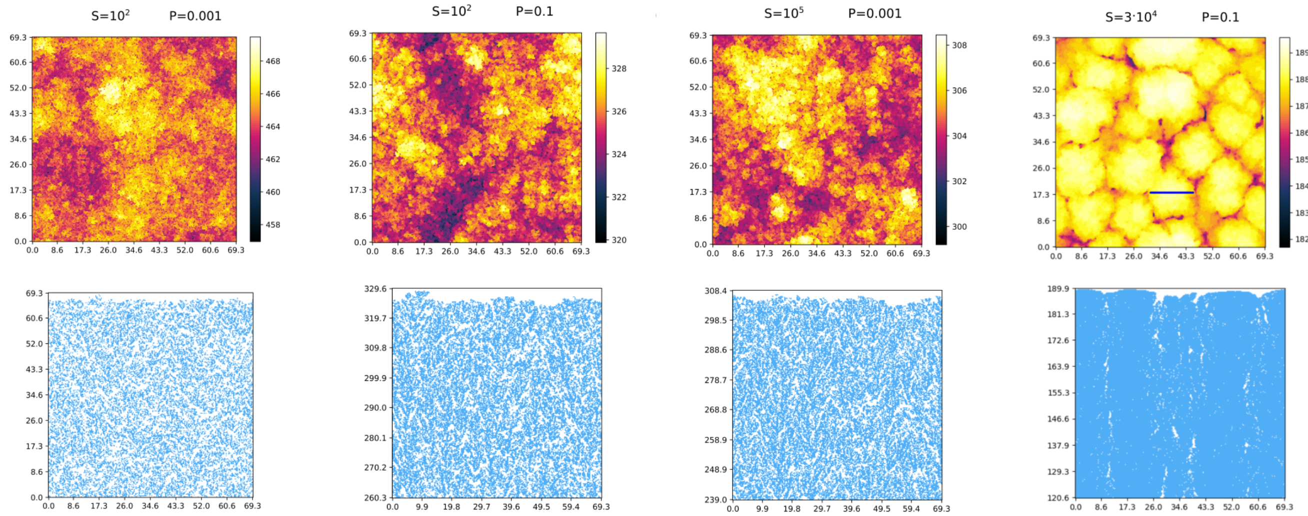

Figure S1: Top and cross-sectional views of pure Si films grown in simulations with the indicated parameters. All lengths are in nanometers.

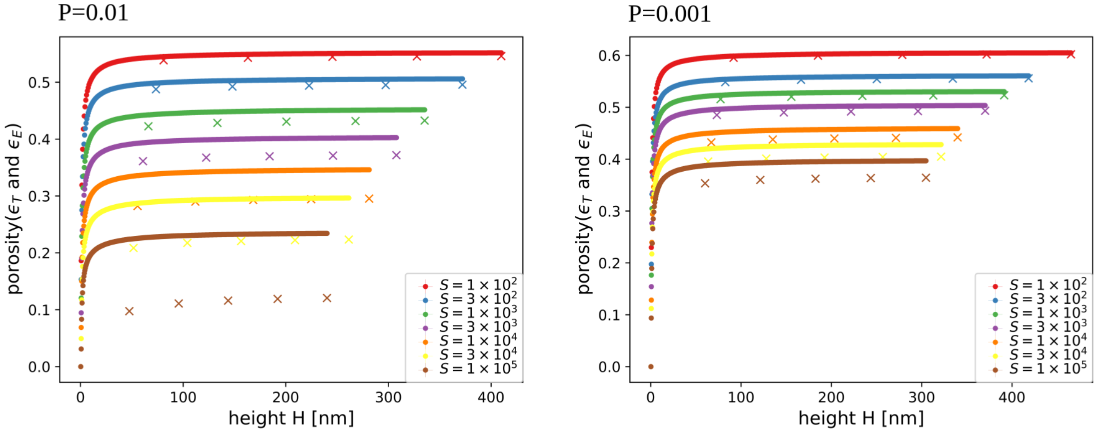

Figure S2: Evolution of total porosity and effective porosity with the thickness of films grown in simulations with for the indicated values of S and P. The uncertainties in the porosity are nearly of the same order as the size of the data points.

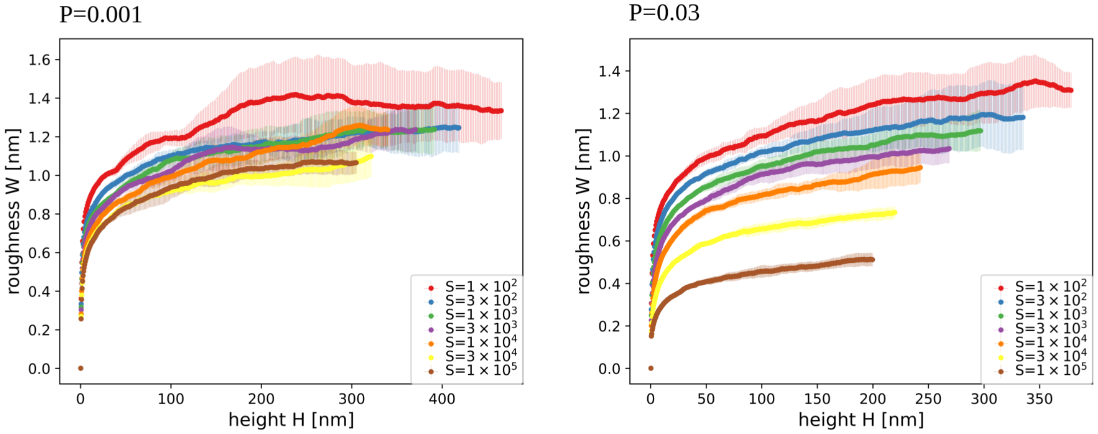

Figure S3: Surface roughness as a function of the thickness of films grown in simulations with the indicated values of S and P. The shadowing shows the uncertainties.

Hybrid films

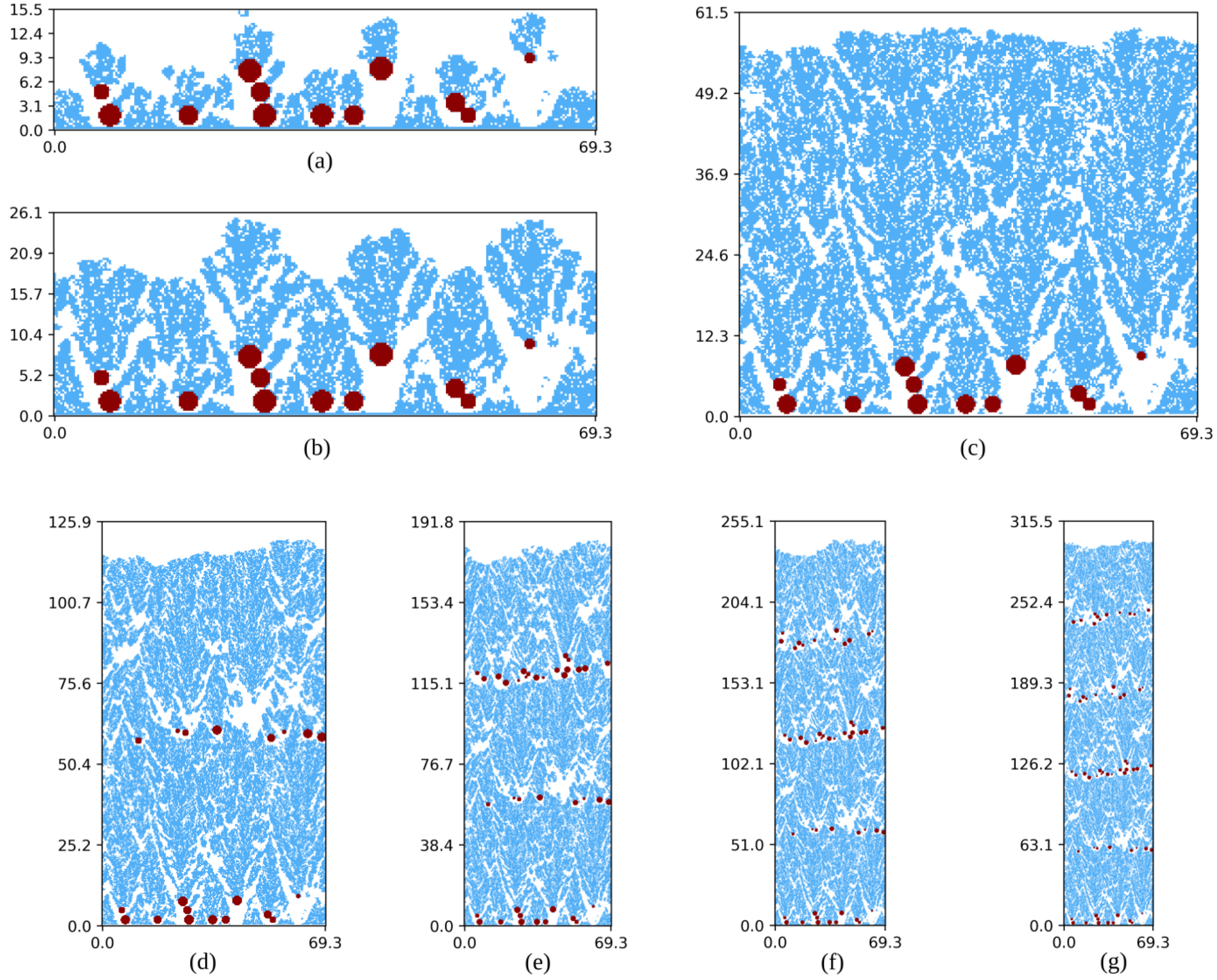

Figure S4: Cross-sectional views of hybrid films grown in simulations with $S = 10^4$ and $P = 0.03$ after the deposition of: (a) one Ta NP scaffold and 0.1 Si layer; (b) one Ta NP scaffold and 0.3 Si layer; (c)-(g) from 1 to 5 pairs of Ta NP scaffolds and Si layers.

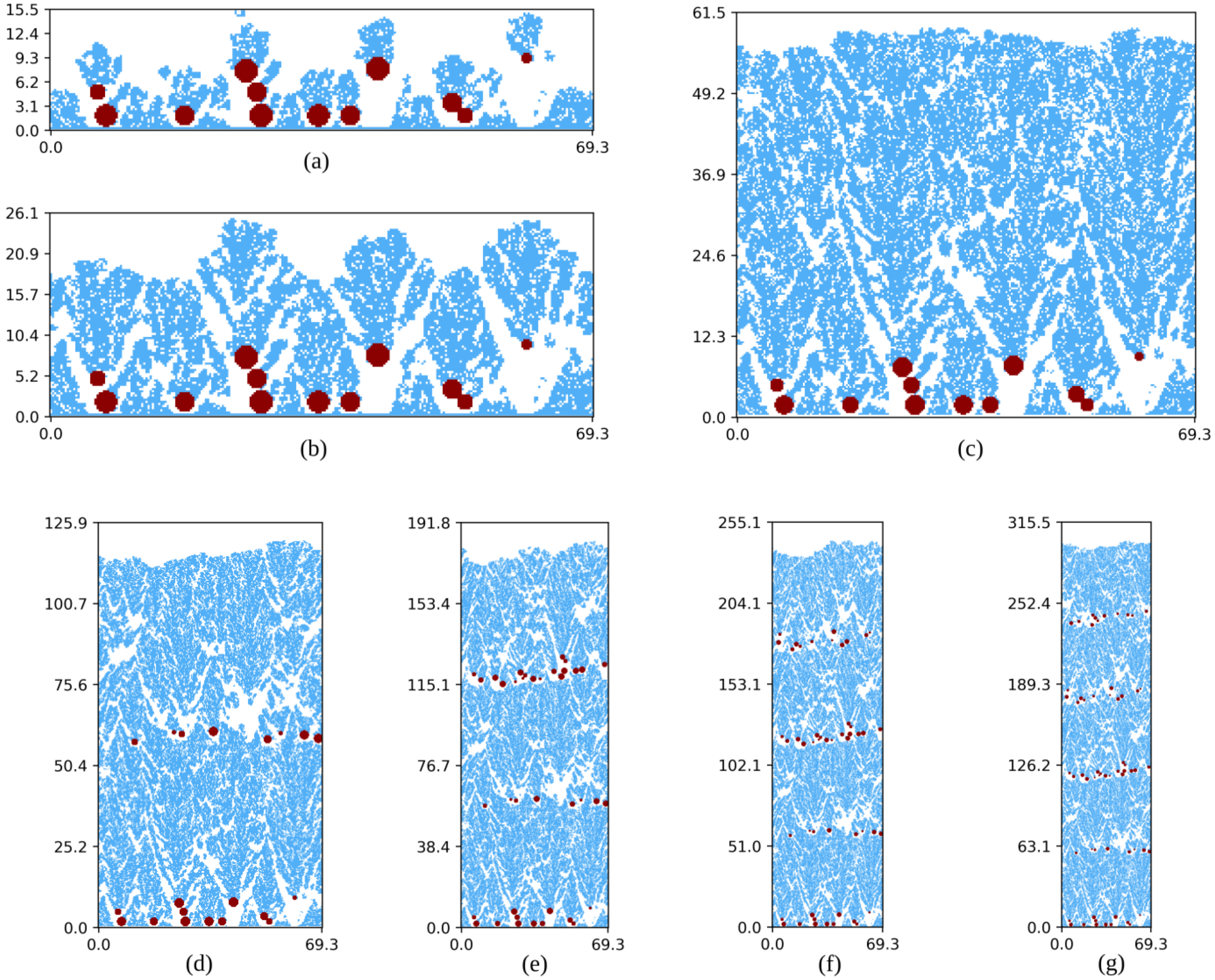

Figure S5: Cross-sectional views of hybrid films grown in simulations with $S = 3 \times 10^4$ and $P = 0.03$ after the deposition of: (a) one Ta NP scaffold and 0.1 Si layer; (b) one Ta NP scaffold and 0.3 Si layer; (c)-(g) from 1 to 5 pairs of Ta NP scaffolds and Si layers.

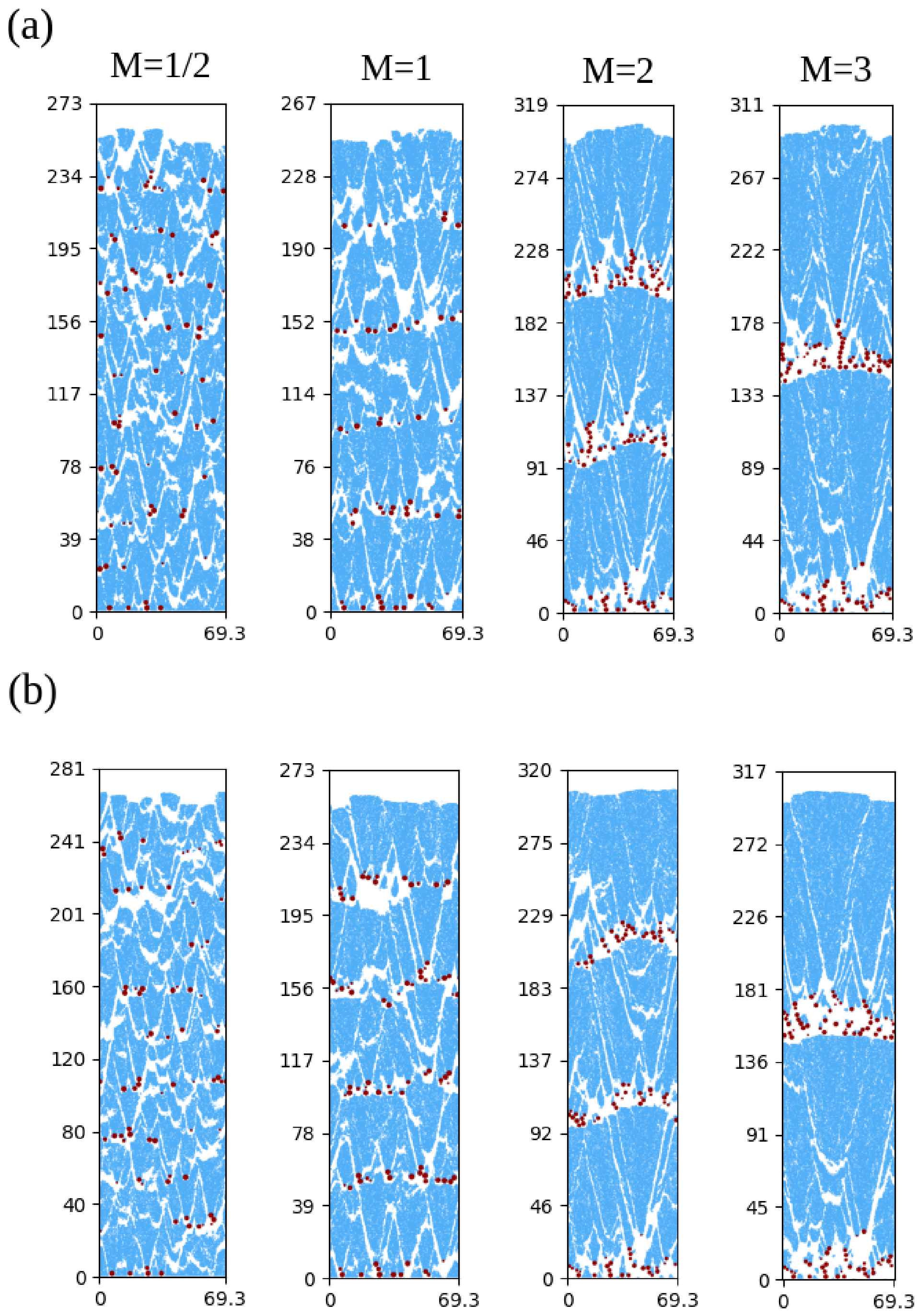

Figure S6: Cross-sectional views of hybrid films grown in simulations with different masses of scaffolds and layers (factors M) and: (a) $S = 10^4$, $P = 0.1$; (b) $S = 10^5$ and $P = 0.03$.
